# Magnetic Nanoparticles in Bone Tissue Engineering

**DOI:** 10.3390/nano12050757

**Published:** 2022-02-24

**Authors:** Akshith Dasari, Jingyi Xue, Sanjukta Deb

**Affiliations:** 1 Faculty of Dentistry, Oral & Craniofacial Sciences, King’s College London, Floor 17 Tower Wing, Guy’s Hospital, London Bridge, London SE19RT, UK; akshith.dasari@kcl.ac.uk (A.D.); jingyi.xue@kcl.ac.uk (J.X.); 2 Faculty of Life Sciences & Medicine, King’s College London, Guy’s Campus, London SE11UL, UK

**Keywords:** magnetic nanoparticles, SPIONs, bone tissue engineering, scaffolds for bone tissue engineering

## Abstract

Large bone defects with limited intrinsic regenerative potential represent a major surgical challenge and are associated with a high socio-economic burden and severe reduction in the quality of life. Tissue engineering approaches offer the possibility to induce new functional bone regeneration, with the biomimetic scaffold serving as a bridge to create a microenvironment that enables a regenerative niche at the site of damage. Magnetic nanoparticles have emerged as a potential tool in bone tissue engineering that leverages the inherent magnetism of magnetic nano particles in cellular microenvironments providing direction in enhancing the osteoinductive, osteoconductive and angiogenic properties in the design of scaffolds. There are conflicting opinions and reports on the role of MNPs on these scaffolds, such as the true role of magnetism, the application of external magnetic fields in combination with MNPs, remote delivery of biomechanical stimuli in-vivo and magnetically controlled cell retention or bioactive agent delivery in promoting osteogenesis and angiogenesis. In this review, we focus on the role of magnetic nanoparticles for bone-tissue-engineering applications in both disease modelling and treatment of injuries and disease. We highlight the materials-design pathway from implementation strategy through the selection of materials and fabrication methods to evaluation. We discuss the advances in this field and unmet needs, current challenges in the development of ideal materials for bone-tissue regeneration and emerging strategies in the field.

## 1. Introduction

Bone tissue is capable of natural regeneration by harnessing intramembranous and endochondral ossification since postnatal bone can carry out self-repair and remodel at the site of damage to restore function [1]. However, this self-healing mechanism fails to occur in the case of critically sized defects [2]. Traumatic injury, degenerative disease, tumour resection, infection and congenital defects can all lead to critical-sized bone defects, which necessitate intervention to achieve complete healing [3]. Bone autografts and allografts as well as metallic implants are presently established treatment modalities for critical-sized defects. Autografts possess excellent osteoinductive, osteoconductive properties that are histocompatibile and non-immunogenic hence resulting in higher rates of success. Nevertheless, autografts can result in significant donor site morbidity and surgical risks such as infection, bleeding and pain, making them less feasible for defects that require large volumes of bone [4]. Using allografts can eliminate the issue of donor site harvesting, however, they have lower osteogenic capability along with concerns of immunoreactions and infection transmission. Both these grafting techniques incur substantially high costs to perform, and the grafting market is struggling to meet the current high demands [5]. Although a wide variety of synthetic biomaterials are used as alloplastic materials, the clinical outcomes are variable. Overall, none of the current treatment options have all the desired characteristics that possess good osteoinductive properties and exhibit angiogenic potential, biosafety, availability, reasonable cost, low patient morbidity and no size restrictions [6].

### Bone Tissue Engineering

Bone tissue engineering (BTE) is a field that strives to outperform current treatments by providing potential alternatives to overcome the limitations of current approaches in bone regeneration [7]. Bone tissue engineering aims to induce new tissue repair and regeneration by the synergy of reparative cells, signalling molecules and scaffolds. Three-dimensional scaffolds that can mimic the extracellular matrix template, whilst offering mechanical support, provides an attractive environment for cell attachment, proliferation and differentiation. Hence, materials for scaffolds are selected based on their ability to display biomimicry, with regards to key parameters such as mechanical properties, porosity, osteoinduction and osteoconduction. Research over the last two decades has focused on scaffolds with and without biological components, however, the complex regulatory requirements, high costs and need for feasible clinical translational technologies have steered the direction of research towards acellular scaffolds that are designed to recruit cells from surrounding native tissue post-implantation to enable bone regeneration. The success of in-situ bone regeneration depends on the effective recruitment of host stem cells or progenitor cells into the scaffold consequently inducing the infiltrating cells into tissue-specific cell lineage for functional bone tissue regeneration. The use of appropriate bioactive agents or bioactive scaffolds that can recruit cells with osteogenic capability through the formation of mineralised matrices through the entire scaffold structure can help drive and accelerate the regenerative process. Crucially, vascularisation must occur alongside bone formation to support the needs of the growing tissue [8,9]. Many strategies have been explored towards enhancing the osteogenic and angiogenic capacity of scaffolds [7,8] with structural hallmarks close to the nanoscale composition of natural bone and modifications to enhance physicochemical interactions, biocompatibility, mechanical stability and cellular attachment/survival. While bone tissue engineering has provided promising results, it has become increasingly clear that the hierarchical integration of bone scaffolds and vascular networks to create constructs that support both osteogenic and angiogenic growth is crucial for success. Leveraging principles that can drive the in-situ body’s innate cellular populations to regenerate tissues is of high interest and with advances in nanomaterials and specifically magnetic nanoparticles (MNP), provide new opportunities in the development of more effective therapeutic efficacy.

This review will begin with a short introduction to magnetic nanoparticles and superparamagnetic iron oxide nanoparticles (SPIONS) and the specific ways in which MNPs can influence bone tissue regeneration. The influence of MNPs on cells, delivery of bioactive agents and the use of MNPs in scaffolds through a critical analysis of some selected studies on bone tissue engineering will be discussed. The review then shifts its attention towards the toxicity concerns related to the use of MNPs in biomedicine by focussing on a group of studies that investigated the impact of MNPs on the osteogenic potential of stem cells to elucidate the role of toxicity in BTE. Overall, the review hopes to provide a critical judgement on the use of MNPs in BTE.

## 2. Magnetic Nanoparticles and Bone Tissue Engineering

### 2.1. Magnetic Nanoparticles

**Figure 1:**
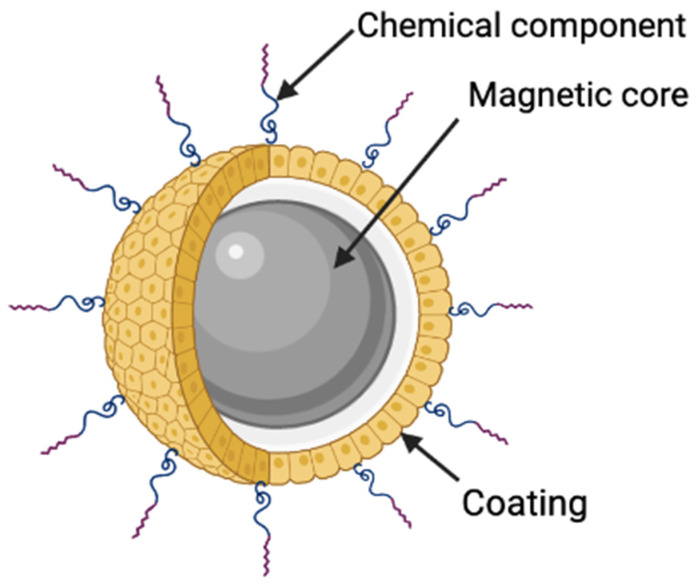
Components of a typical MNP.

**Figure 2:**
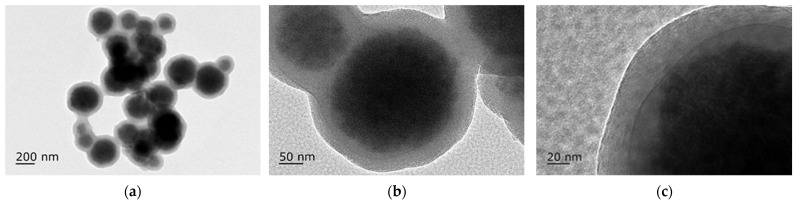
(**a**) TEM image of core-shell Fe_3_O_4_-SiO_2_ magnetic nanoparticles with good dispersity; (**b**,**c**) high-resolution TEM images of core-shell Fe_3_O_4_-SiO_2_ magnetic nanoparticles with obvious Fe_3_O_4_ core and silica layer. (unpublished data, Jingyi Xue, 2022).

Magnetic Nanoparticles (MNPs) are a class of nanomaterials that are composed of metals such as iron or cobalt, which endows magnetic manipulation under the influence of an external magnetic field [10]. They typically consist of a magnetic core and a biocompatible capping along with an optional coating that can provide added functionality (Figure 1). The excitement of MNPs stems from their high modifiability with regards to their size, shape and coating. This means that their physicochemical properties can be specifically tuned towards the desired application, offering versatility to the field of biomedicine [11,12]. Furthermore, their intrinsic biocompatibility has led to MNPs being used in a variety of diagnostic and therapeutic applications [13,14]. These include, use as contrast agents in MRI, magnetically targeted drug delivery and magnetic hyperthermia to note a few. Interestingly, iron-oxide-based MNPs that are sufficiently small can display magnetic behaviour in the absence of an external magnetic field, a phenomenon known as super paramagnetism. Such MNPs are known as Superparamagnetic Iron Oxide Nanoparticles (SPIONs), which have gained the spotlight in biomedicine [15]. SPIONs have high saturation magnetisation and magnetic susceptibility, are chemically stable, biocompatible, biodegradable and non-toxic in nature. Iron oxide nanoparticles can be synthesised with ease and many different approaches have been reported, which include hydrothermal, solvothermal, sol-gel methods, co-precipitation, microwave-assisted, chemical vapour deposition, electrochemical, laser pyrolysis techniques and biosynthesis [16,17]. For biomedical applications, magnetic nanoparticles of iron oxide with the crystal structure of maghemite and magnetite are the most explored, and the temperature and pressure of the reaction conditions are mainly used to control the size and morphology of these MNPs. Since iron oxide MNPs tend to aggregate due to their high surface energy, surface coatings are applied to stabilise the particles and functionalised with various coatings such as chitosan, silica, polyethylene glycol, dextrans, etc. to form a shell. Although the surface of MNPs is relatively inert, which prevents the formation of strong covalent bonds with molecules for functionalisation, the reactivity can be improved through coatings which can then be used to modify the surface. Figure 2 shows the TEM image of an iron oxide nanoparticle with a silica shell created to enhance the reactivity of the core. The ability to manipulate the physicochemical properties of MNPs such as size, shape, morphology, hydrophilicity, surface modification and functionalisation make them powerful components when combined with scaffolds for the development of engineered nanostructures for tissue engineering.

### 2.2. The Influence of Magnetic Nanoparticles on Bone Tissue Engineering

MNPs are fast becoming a key instrument in bone tissue engineering. The literature has evidenced their ability to augment all three components of BTE, being cells, bioactive agents and scaffolds, in a variety of ways. SPIONs have been harnessed for cell induction purposes because of the ability of each nanoparticle to ingenerate an intrinsic magnetic field. The nanoparticles can be internalised by cells, where their magnetism can stimulate them, by promoting the activation of intracellular pathways that facilitate osteogenesis [18,19]. Moreover, a magnetic microenvironment is produced within scaffolds loaded with SPIONs. The surrounding magnetism can elicit changes to ion channels and receptors on the cell membrane which detect this, resulting in significant improvements to osteogenic differentiation and proliferation. Besides this, SPION incorporation can promote cell adhesion with a more conductive scaffold microarchitecture, whilst bolstering the mechanical properties of the scaffold. SPIONs could potentially have a role in enhancing angiogenesis within a scaffold and the inclusion of an external magnetic field can amplify this further. Overall, the addition of SPIONs in scaffolds could overcome the main challenge for BTE, which is producing a mechanically strong scaffold with excellent bone-forming capacity that achieves vascularisation and integrates with host tissue.

The use of an external magnetic field to control the movement and function of MNPs can diversify the applications of MNPs in BTE. The combination of an oscillating external field and MNPs within a defect could augment cell induction and remotely deliver biomechanical stimuli at the cellular level to enhance osteogenesis. Furthermore, MNPs can effectively transport certain bioactive agents or mesenchymal stem cells under the influence of an external magnetic field to a bone defect site. This guiding technique enables localised delivery and retention that could maximise bone regeneration therapy whilst minimising side effects to surrounding tissues. A novel method for cell-based tissue engineering has adopted the use of an external magnetic field to precisely control and manipulate cells that have internalised MNPs. This fabrication technology could offer a more practical solution over other methods and produce a thick, mechanically strong pre-vascularised construct for BTE.

#### 2.2.1. Magnetic Nanoparticles and Cells

MNPs hold the potential to conduct cellular and molecular level interactions, consequently influencing cellular function. Specifically, studies have highlighted the ability of SPIONs to enhance osteogenesis within stem cells in the absence of an external magnetic field [18,19,20].

##### Cell Induction

**Figure 3:**
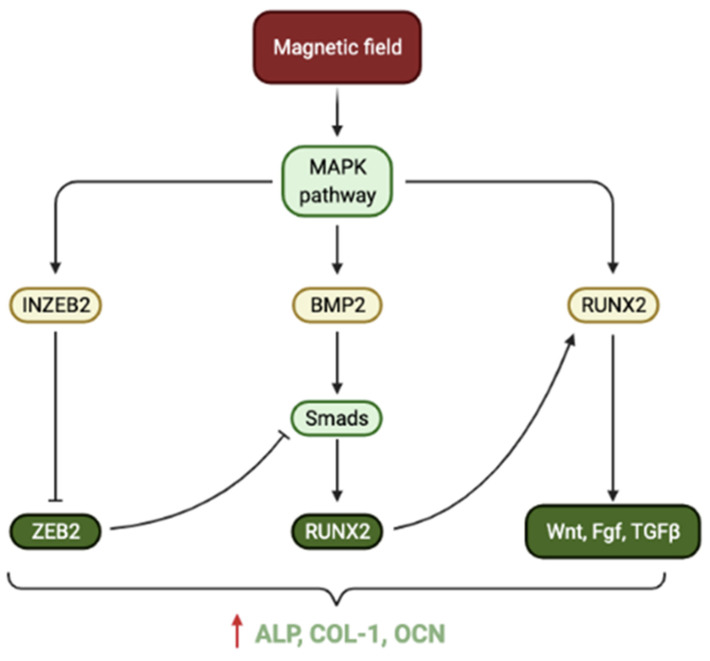
Schematic illustration of how the magnetic field generated from SPIONs can enhance osteogenic differentiation of stem cells through the classical Mitogen-activated protein kinase (MAPK) pathway.

Mesenchymal Stem Cells (MSCs) can be impacted by physical or biochemical stimuli originating from the intracellular or extracellular microenvironment [21,22]. Stimuli can be sensed by receptors within the cell membrane or the cytoskeleton, which then elicit chromosomal responses that influence protein synthesis and gene expression [23,24]. These genetic responses to stimuli are vital to modulate the differentiation pathway of MSCs. SPIONs can initially provide mechanical stimulation to MSCs upon direct interaction with the cell membrane [25]. They subsequently can become internalised within MSCs by endocytosis and produce further stimulation within the cell. Each SPION can generate an intrinsic magnetic field, which is responsible for cell stimulation. The classical Mitogen-activated protein kinase (MAPK) pathway is activated upon such stimulation, leading to the upregulation of downstream genes that are associated with osteogenesis [20]. Firstly, the overexpression of RUNX2, a marker of early osteogenic differentiation, has a complex involvement in multiple major signalling pathways that promote osteogenesis [26]. The upregulation of BMP2 can activate Smads proteins, which results in the expression of yet more RUNX2. This summarises the activation of the BMP2/Smads/RUNX2 signalling pathway, which plays a crucial role in bone morphogenesis [27]. Finally, the upregulation of INZEB2 is pivotal in the maintenance of osteogenesis because it downregulates ZEB2, a factor that suppresses the BMP2/Smads/RUNX2 signalling pathway [28]. Overall, these processes work to significantly amplify ALP, collagen type 1 and osteocalcin expression at the mRNA and protein levels highlighting the impact of MNPs on osteogenesis (Figure 3).

SPIONs have also been reported to accelerate cell growth of MSCs once within the cell and therefore improve proliferation rates [29]. One mechanism by which this occurs involves the intrinsic peroxidase-like activity of SPIONs by diminishing intracellular hydrogen peroxide, a reactive oxygen species that can inhibit cell proliferation [30]. An additional mechanism involves the lysosomal degradation of MNPs, which produces free iron ions. Excess iron ions can alter the expression of various protein regulators of the cell cycle, further promoting cell cycle progression and cell growth. However, the biosafety of excess intracellular iron ions must be thoroughly investigated due to concerns of toxicity (discussed in Section 4).

MSCs crucially require biomechanical stimuli to drive differentiation and subsequent osteogenesis at a defect site. A technology that combines the use of MNPs and an external magnetic field known as MICA (Magnetic Ion Channel Activation) [30] has been shown to provide a biomechanical stimulus by specifically targeting mechanosensitive ion channels in the MSC cell membrane, which activates mechano-transduction pathways, thereby promoting bone augmentation. Early reports indicate that remote control of this signalling process using MICA has the potential to both drive and regulate tissue regeneration and healing [31,32].

##### Cell Guidance

A fundamental challenge for cell regeneration therapies is achieving homing and retention of cells at the site of injury. Strategies that enable cell guidance tend to improve the efficiency of cell delivery to maximise therapeutic effects whilst minimising dispersion to surrounding tissues and the side effects that may occur. MNP-based cell targeting systems offer promise with potential application in bone regeneration. The homing capacity of MSCs labelled with SPIONs have been determined by the expression of specific chemokine receptors that potentiate the migration of MSCs to an injury site. CXCR4, CCR1 and c-Met are examples of these migration-related receptors, which were all upregulated in labelled MSCs. This was supported by an in-vitro study in which the labelled MSCs displayed greater migration potential, suggesting that SPIONs could enhance the migratory capacity to an injury site [33]. This was further confirmed by an in-vivo study using an inflammatory rat ear model where intravenous injections of labelled MSCs showed the greatest MSC translocation and accumulation at the inflamed site, uniquely in the absence of an external magnetic field [33]. Because sites of bone trauma exhibit pro-inflammatory conditions, regeneration strategies that employ MSCs could also adopt SPIONs to improve the migration and retention properties of MSCs.

The presence of an external magnetic field provides supplementary control over the movement of MNPs and can therefore be more effective than the use of just MNPs in achieving cell homing. In support of this finding a porous interconnected hydroxyapatite ceramic scaffold when used to bridge rabbit ulnar defects exhibited a significantly greater number of labelled MSCs throughout, which received external magnetic control in comparison to the group that received only percutaneously injected SPION-labelled MSCs [34]. The study also showed that a short external magnetic stimulation was sufficient to achieve greater cell retention, consequently, exhibiting superior healing outcomes than the group that received labelled MSCs alone.

Cell-based therapies that involve direct injection of cells in a defect site is typically suited to minimally invasive regenerative treatments, such as intraarticular injection of MSCs in cases of severe osteochondral defects. However, the efficacy is limited due to the wide dispersion of MSCs within the joint and consequent scar tissue formation. An in-vivo study investigated [35] the use of SPION-labelled MSCs with a short exposure to an external magnetic field using a rabbit osteochondral defect model to enhance the efficacy of the intraarticular injection for osteochondral defects. The study identified no issues of dispersion or scar tissue formation along with complete healing of subchondral bone covered by a layer of hyaline cartilage. The external magnetic field was able to retain the labelled cells in the desired location, which achieved localised delivery that reduced the impact on surrounding tissues whilst maximising the therapeutic effect at the desired location. Furthermore, this magnetic targeting method used a lower dose of MSCs compared to other studies focusing on the repair of similar defects [35]. Overall, this study encompasses the benefits of a magnetic targeting system. Magnetic targeting has the potential to greatly enhance the efficacy of cell-based regenerative treatments that involve direct stem cell injection, which hold a clear advantage over more invasive and complex surgical alternatives.

##### Cell-Based Tissue Engineering

**Figure 4:**
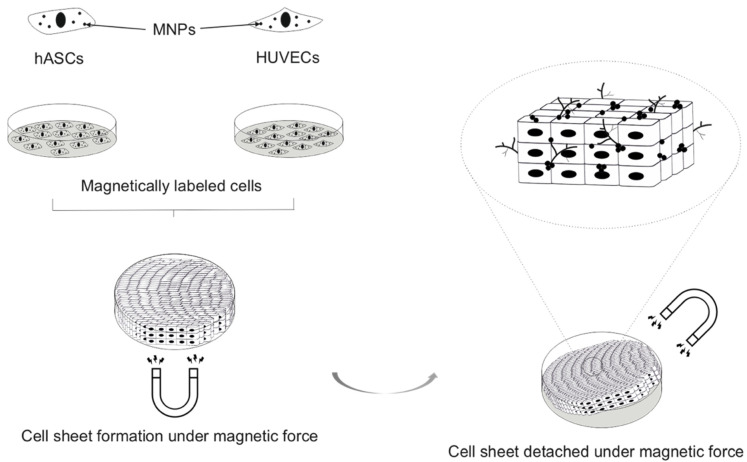
Schematic displaying organisation of MNP-labelled cells into cell sheets and assembly into multilayer constructs in presence of an external magnetic field.

Cell-based technology allows for perseveration of the endogenous ECM whilst avoiding the limitations of scaffolds such as rejection and tissue failure [36,37]. However, engineering bone tissue requires thick constructs with sufficient mechanical strength, which is challenging with cell-based technologies because of tissue ischaemia in the inner cell mass. Cell sheet technology has been proposed as a method for creating thick constructs that have vascularisation potential, although layering individual cell monolayers and cell positioning has proven to be arduous and time-consuming [38]. MNP technology limits these fabrication barriers and holds promise in delivering cell-sheet technology compatible with BTE requirements. Specifically, cells internalise the MNPs, and these cells can then be attracted to a culture dish floor using a magnetic force. Further cell layers can be similarly added to create multilayer thick constructs (Figure 4). This technology is simple, cost-effective and time-saving and also enables precise control of the shape of cell sheets with magnet arrangement. It can promote cellular interactions, allowing for cell adhesion, cell-cell junction formation and ECM deposition.

Harnessing this technology, Silva et al. [39] attempted to produce a pre-vascularised cell sheet construct using adipose-derived stem cells (ASCs) and human umbilical vein endothelial cells (HUVECs) in triple sheet layered confirmations (ASC/HUVEC/ASC) suitable for bone regeneration. This technique generated homotypic cell interactions as well as heterotypic interaction between ASCs and HUVECs. Heterotypic interactions lead to a synergistic effect that promotes the self-generation of vital growth factors such as BMP-2 and VEGF. The validated cross-talk between these two growth factors triggered both angiogenesis and osteogenesis in-vitro. Additionally, this pre-vascularised hierarchical 3D construct demonstrated high angiogenic potential in-vivo [39]. This technology potentially opens an avenue for scaffold-free BTE, however higher costs, complexity and mechanical concerns make scaffolds based BTE more favourable [40]. Another approach that has been reported is applying cell surface engineering wherein MNPs are not internalised by cells and instead are immobilised on cell membranes [41,42]. Cells are rendered magnetically responsive using polymer modified stabilised MNPs and magnetic force-based tissue engineering employed to fabricate viable cell sheets and 3D multicellular spheroids [42,43]. This process is reported to be an efficient and quick process with lower toxicity to cells.

##### Delivery of Bioactive Agents Using Magnetic Nanoparticles

Targeted and controlled delivery of bioactive agents in specific sites especially at a cellular level can fortify bone regeneration whilst limiting associated side effects. Bioactive agents including growth factors, drugs, genes, etc. are known to positively influence bone regeneration, thus using MNPs as carriers for such agents provides an additional advantage due to the ability to stimulate them under magnetic fields.

A porcine kyphoplasty model was reported to show that a magnetic bone cement injected to manage vertebral compression fractures (VCFs) was able to localise systemically injected MNPs at the vertebral body where the cement was injected [44]. Therefore, it was proposed that this method could be used as an effective targeted drug delivery system for the treatment of osteoporosis induced VCFs [44]. Interestingly, MNPs can potentially deliver curative osteogenic drugs such as simvastatin [45] or bone morphogenic protein [44], a potent osteoinductive growth factor, meaning that this targeting system can be tailored as a bone regenerative treatment for osteoporosis. This clearly has an advantage over therapeutic osteoporosis treatments, which can result in low blood supply to the spine and systemic toxicity of drugs that deposit in healthy tissues.

Besides targeted delivery, the stability of a bioactive agent can be optimised with conjugation to MNPs. The covalent conjugation of fibroblastic growth factor (FGF) to human serum albumin (HSA)-coated MNPs were reported to significantly enhance the stability of FGF in tissue cultures resulting in greater biological efficacy of the growth factor [46]. Once internalised by human BMSCs, conjugated FGF accentuated the proliferation and osteogenic differentiation capacity of the cells to a greater extent in comparison to free FGF. Stabilising growth factors by conjugation to MNPs reduces its susceptibility to enzymatic degradation and inhibitors, which improves its availability to target cells in bone regeneration [46].

Magnetic patterning can also provide a simple and rapid method for creating a growth factor concentration gradient that could replicate the complex hierarchical microstructure of physiological tissues. Since osteochondral tissue engineering demands the gradual transition between bone and cartilage, systems that create a smooth gradient distribution of growth factors within a hydrogel can effectively recapture this transition, although they carry the burden of complex fabrication. To ease the gradient fabrication process, a study reported [47] forming BMP-2 conjugated to glycosylated SPIONs, that were magnetically aligned in a gradient fashion within an agarose hydrogel seeded with human MSCs. The glycosylated SPIONs guarded BMP-2 against degradation and enabled sustained diffusion-dependent release that spatially mediated osteogenesis. The gradient of BMP-2 produced mineralised bone where the growth factor concentration was highest, which smoothly transitioned to cartilage as the concentration decreased, resulting in structurally robust osteochondral tissue. Hence, this example of a highly controlled bioactive agent delivery and release within a construct is of interest.

## 3. Magnetic Nanoparticles in Scaffolds for Bone Tissue Engineering

Many different strategies have been investigated to combine scaffolds, cells and biologically active cues using a wide range of fabrication techniques, to provide innovative solutions for bone tissue biomimicry. Thus far there has been an emphasis on the microarchitecture of the scaffold that focuses on porosity, pore size and pore interconnectivity, to facilitate the proper mass transfer of nutrients and waste products, as well as vascularisation and tissue infiltration in addition to mechanical properties and bioactivity. Mechano-transduction is understood to facilitate osteogenesis and thus has been considered into a multitude of in vitro bone tissue engineering approaches to effectively control cell behaviour. More recently, magnetic nano actuation is being explored to remotely manipulate cell behaviour with much greater control and accuracy. MNPs can be integrated within scaffold matrices using a range of fabrication techniques such as electrospinning, covalent linkages and freeze-drying. The effect of MNPs containing scaffolds on osteogenesis is discussed first and then their role in promoting angiogenesis is examined.

### 3.1. Impact on Osteogenesis

A summary of the findings of selected recent studies that investigated the impact of incorporating MNPs into scaffolds on osteogenesis is presented in Table 1. It is important to note that the studies included in this table used different types of scaffolds with differing chemistries, variation in MNP content and magnetic intensity. Despite these differences, all studies concluded that the addition of MNPs significantly enhanced osteogenesis. There was also a substantial crossover in the mechanisms suggested for the enhanced osteogenic activity between the studies.

Most of the results summarised in Table 1 also indicated that bioactivity was enhanced in the presence of MNPs in scaffolds, specifically revealing greater cell adhesion and cell spreading, which displayed more stretched and spindle-like morphology [48].

One reason for these findings could be attributed to the greater hydrophilicity of the scaffolds since MNPs are inherently hydrophilic and their incorporation remarkably improves the wettability of the scaffold thereby enhancing the affinity for cells and proteins that mediate cell attachment [49]. The incorporation of MNPs was also reported to alter nano structural features of scaffolds, especially in calcium phosphates (CPC) wherein the crystal size shows a decrease leading to a greater surface area [50,51], consequently increasing adhesion of protein molecules that facilitate subsequent cell adhesion. Similar findings were observed on polycaprolactone (PCL) polymeric scaffolds that exhibited enhanced protein adsorption which increased with increasing MNP content [49]. Additionally, MNPs induce changes in nanostructure topography, such as greater surface roughness, which encourages cell adhesion and spreading. Overall, there is consensus on the relationship between MNP incorporation in scaffolds and enhanced cell adhesion mainly attributed to increased hydrophilicity [48,49,52,53,54] or the nanostructure [50,51,52,53,55].

The inclusion of MNPs and its concentration has a bearing on the mechanical properties of scaffolds. Several studies [51,53,54,55,56] have reported that improvement in mechanical properties is dependent on the amount of MNPs present and beyond certain concentrations show a steady decline in their mechanical properties. The improvement in mechanical properties [48,52] has been attributed to either chemical interactions or the influence on microarchitecture. For example, PCL-MNP scaffolds have been reported to exhibit an increase in stiffness [56] whilst a chitosan collagen scaffold showed a higher compressive modulus due to the interactions between the inorganic MNPs and organic chitosan collagen matrix [55]. In contrast, improvements in the mechanical properties of CPC-based scaffolds were mainly attributed to a reduction in pore size and pore volume fraction [50,52] although some studies have demonstrated that MNP incorporation has no significant impact on the porosity level of a scaffold [49,54,57,58]. However, it is prudent to consider the material type to understand the effect on porosity since MNPs have been found to generally reduce the porosity of PCL or CPC scaffolds, whilst having the opposite effect on natural scaffold materials such as chitosan or collagen. From the analysis of these studies, we can deduce that the mechanical properties of scaffolds can be manipulated by incorporating MNPs; however, the optimisation of the content is imperative to achieve benefits.

A common feature of the different studies summarised in Table 1 was the reportedly remarkable improvement in cell proliferation and osteogenic differentiation irrespective of the type of scaffold. Most of the studies utilised SPIONs such as magnetite or maghemite as the MNP and their addition endowed the scaffolds with a superparamagnetic property. Each SPION within the scaffold behaves as a single magnetic domain and the combined effect of all the nanoparticles generates a magnetic microenvironment. The cells are stimulated by this microenvironment because of significant alterations to ion channels and receptors on the cell membrane that activate intracellular signalling pathways [49,53,54]. This effect is very similar to cell responses to mechanical stimuli whereby cells are transduced via the activation of mechanosensitive ion channels or receptors [59]. Magnetic induction could therefore explain the accelerated cell cycles and osteogenic differentiation, although the exact mechanism by which this occurs is yet to be elucidated. In contrast, Xia et al. used a demagnetised magnetic scaffold generated through high-temperature annealing and compared directly to magnetic scaffolds, which revealed no difference in cell behaviour [50]. Therefore, the study excluded the effect of magnetism and instead concluded that the nanostructure was the main reason for improved cell performance. Hence, further work needs to be conducted to authenticate the role of magnetism in osteogenesis.

Literature findings suggest that MNPs can induce a transmembrane effect in the form of an upregulated magneto-sensing receptor that promotes osteogenesis within ADSCs, however, the signalling cascade that mediates this is not clear. Chen et al. [48] found that the gene expression of an exogenous magnetoreceptor, iron-sulphur cluster assembly protein 1 (ISCA1), was upregulated because of the magnetic microenvironment in ADSCs. Moreover, the expression of ISCA1 was highly correlated with the upregulated expression of osteogenic genes ALP and RUNX2. Xia et al. [52] proposed that the WNT signalling pathway regulated the osteogenic differentiation of DPSCs. The upregulation of the transmembrane receptor WNT1 and intracellular protein *β* catenin indicates the role of this pathway in mediating osteogenic gene expression [52]. Alternatively, Lu et al. [56] observed that the BMP-2/Smad/RUNX2 pathway was activated within BMSCs upon magnetic stimulation, indicated by the greater expression of its components [56]. These contrasting findings may imply that the signalling pathway via which proliferation and osteogenic differentiation occur is dependent on the stem cell type being investigated. However, the studies did not rule out the involvement of other signalling pathways, meaning that multiple pathways could be working simultaneously. Hence a study that investigates the involvement of a range of signalling pathways in ADSCs, DPSCs and BMSCs is needed to elucidate how different stem cells mediate osteogenesis upon magnetic stimulation.

As noted earlier that the concentration of MNPs in a scaffold influences mechanical properties, it too has a profound effect on cell proliferation and osteogenic differentiation. Although the magnetic microenvironment can be intensified by increasing the MNP content in scaffolds [48] there is a limiting value after which cell activity markedly decreases. Studies that assessed different content of MNP noted that cell proliferation and osteogenic differentiation increased with increasing MNP content [49,53,56,60]; however, in both PCL and CPC scaffolds, cell performance, including proliferation and ALP activity, was found to be at its maximum at 3% and 15% MNP content, respectively, [53,56] after which there was rapid decline. These findings indicate that increasing the MNP content can improve cell performance, however, the optimum content must be carefully elucidated to avoid toxicity.

In vivo studies to validate the potential of MNP-loaded scaffolds, refs. [54,55,56,57,60,61,62] showed superior bone formation and mineralisation of defects filled with MNP-loaded scaffolds compared to controls. This was quantitatively represented with significantly greater bone mineral density and bone volume fraction. A study by Zhao et al. [55] showed that osteoblasts had greater adhesion and infiltration through the scaffold, supporting the hypothesis that MNPs improve the nano structural properties [55]. It was also reported that the new bone tissue was well fused and better integrated with the host bone than in control groups [56,60]. Overall, the subjects that received MNP-loaded scaffolds displayed better healing outcomes.

**Table 1:** A table summarising the various studies that investigated the impact of MNP-incorporated scaffolds on osteogenesis.

Scaffold Material	MNP Composition	MNP Content within Scaffold	Magnetism Intensity (emu/g)	Osteogenic Impact	Mechanism
HA and Collagen [61]	NI	2.65%	NI	Enhanced bone maturity in-vivo, identified by improved mechanical properties.	Incongruous magnetic moment created by the distribution of MNPs within the scaffold.
PCL [59]	Maghemite	7.9%	NI	Improved cell adhesion, proliferation and osteogenic differentiation (elevated ALP) of MSCs.	MNP incorporation generates a magnetic microenvironment.
PCL [52]	GdHA	2.67%	NI	Greater cell attachment, spreading, proliferation and osteogenic differentiation (higher ALP, RUNX2) of MSCs. Improved mechanical properties.	Gadolinium released entered cells and promoted cell cycle progression. Greater hydrophilicity and surface area facilitate protein adsorption. Reduced PCL fibre diameter increases scaffold strength.
PCL [62]	FeHA	4.5%	NI	Improved cell growth. Scaffold filled with new bone after just 4 weeks in-vivo.	MNP incorporation generates a magnetic microenvironment.
PCL [49]	Magnetite	5% 10%	5%—1.6 10%—3.1	Greater cell adhesion, proliferation and osteogenic differentiation (enhanced cellular mineralisation) of MSCs.	Elevated hydrophilicity improved cell adhesion that facilitated proliferation and differentiation to follow. MNP incorporation generates a magnetic microenvironment.
PCL [56]	Magnetite	5%, 10%, 15%, 20%	5%—1.0 20%—11.2	Better cell adhesion, spreading, penetration and osteogenic differentiation (ALP, COL-1, OPN, BSP) of MSCs. Histology showed higher blood vessel formation and better integration with the host tissue in-vivo. Enhanced mechanical properties.	MNP incorporation generates a magnetic microenvironment. Controlled degradation rate allows ingrowth of cells and vascularisation. Strong chemical interaction between MNPs and polymer chains.
PCL and PLGA [48]	Maghemite	16.4%	3.56	Improved cell adhesion, spreading and osteogenic differentiation (higher ALP, RUNX2, OCN, COL-1 and bone mineralisation) of ADSCs. Better mechanical properties.	Greater hydrophilicity and protein adsorptions facilitate cell attachment. Higher gene expression of a transmembrane magnetoreceptor ISCA1-osteogenic enhancement as a result of transmembrane effect of MNPs.
PLLA and PGA [60]	Magnetite	2.5%, 5%, 7.5%, 10%	2.5%—1.66 10%—8.51	Greater cell adhesion, spreading, proliferation and osteogenic differentiation (ALP) of MG63 cells. Improved mechanical properties. Better BMD, BVF, fusion and blood vessel formation in-vivo.	Improved hydrophilicity and magnetic microenvironments facilitate improved cellular activity. MNPs resist deformation of the polymer chains. Microenvironment promoted adhesion, migration and differentiation of osteocytes in-vivo.
PCL and Mesoporous Bioactive glass [58]	Magnetite	5%, 10%, 15%	5%—3.1 10%—6.2 15%—9.3	Increased cell adhesion, proliferation and osteogenic differentiation (elevated ALP, RUNX2, OCN, BMP-2 and COL-1) of MSCs.	Improved hierarchal pore structure. MNP incorporation generates a magnetic microenvironment.
CPC [51]	Magnetite	0.05–5%	0.1%—0.05 1%—0.35	Greater cell adhesion, spreading, proliferation and osteogenic differentiation (increased ALP) of BMSCs. Improved mechanical properties.	Altered surface morphology- change in crystal shape and reduced size increased the surface area for adhesion of proteins involved in cell adhesion. MNP incorporation generates a magnetic microenvironment.
CPC [50]	Maghemite	NI	NI	Enhanced cell attachment, spreading, proliferation and osteogenic differentiation (increased ALP, RUNX2, OCN, COL-1) of DPSCs.	Altered surface morphology-reduced crystal size increased the surface area for adhesion of proteins involved in cell adhesion. MNPs released by the degrading scaffolds and interact with cells via membrane adsorption and internalisation.
CPC [53]	Maghemite	1–6%	NI	Improved cell adhesion, spreading, proliferation and osteogenic differentiation (increased ALP, RUNX2, OCN, COL-1) of DPSCs. Enhanced the mechanical properties.	Greater hydrophilicity and improved nanostructure facilitated cell adhesion and spreading. The WNT signalling pathway is activated and mediates proliferation osteogenic differentiation upon magnetic stimulation. Cells internalise released MNPs.
Gelatin and Siloxane [54]	Magnetite	1–3%	1%—0.24 3%—0.64	Greater cell adhesion, proliferation and osteogenic differentiation (greater ALP and mineralisation) of MSCs. Improved mechanical properties.	Improved hydrophilicity allowed better cell adhesion. MNP incorporation generates a magnetic microenvironment.
Bioglass and Chitosan [57]	SrFe_12_O_19_	1:7, 1:3 (ratio of SrFe_12_O_19_ to Bioglass)	1:7–4.44 1:3–7.68	Enhanced cell adhesion, spreading, proliferation and osteogenic differentiation (increased ALP, RUNX2, OCN, COL-1, BMP-2) of BMSCs. Greater bone mineralisation, BMD and BV/TV in-vivo.	Proliferation and osteogenic differentiation are mediated by BMP-2/Smad/RUNX2 pathway upon magnetic stimulation.
Chitosan and Collagen [55]	Magnetite	NI	0.025	Improved cell adhesion, proliferation and osteogenic differentiation (better mineralisation) in pre-osteoblasts. Enhanced bony ingrowth, BMD and BVF in-vivo. Better mechanical properties.	Improved hierarchical nanostructure- surface roughness and interconnected porosity. This can improve cell adhesion, cell penetration as well as nutrient transfer and flow transportation in the scaffold.

Abbreviations: NI—Not Included, HA—Hydroxyapatite, PCL—Polycaprolactone, PLGA—Poly(lactic co-glycolic acid), PLLA—Polylactic acid, PGA—Poly(glycolic acid), CPC—Calcium Phosphate cement, GdHA—Gadolinium-doped Hydroxyapatite nanoparticles, FeHA—Iron-doped Hydroxyapatite nanoparticles, BMD—Bone Mineral Density, BVF—Bone Volume Fraction, BV/TV—Bone Volume/Tissue Volume. Magnetite—${\mathrm{Fe}}_{3}O_{4}$; Maghemite—${\gamma \mathrm{Fe}}_{2}O_{3}$.

### 3.2. Effect of MNPs on Angiogenesis

Angiogenesis alongside osteogenesis is pivotal for the survival of cells, especially in deeper regions of a scaffold where the nutrient exchange is even more challenging. This has currently limited BTE to small constructs that do not meet the clinical demands for repairing large bone defects, marking angiogenesis as a major challenge in BTE [63]. To combat this, various strategies to enhance the angiogenic capacity of a construct have been studied [64]. Examples include the co-delivery of growth factors VEGF and BMP-2 [65] or the addition of trace elements such as Mg^2+^ or Si^4+^ [66]. Recent evidence suggests that MNPs may not just influence osteogenesis but also promote angiogenesis implying that MNP-incorporated scaffolds could potentially have a dual function. The promotion of both processes is termed osteogenic-angiogenic coupling, which plays a major role in bone regeneration.

To elucidate the effect of MNPs on angiogenesis, several studies have been attempted to understand the effect. An in vivo study implanted gelatine sponges with SPIONS in incisor sockets of rats that exposed the SPIONS due to the rapid degradation of the gelatine [19]. The gelatine sponges carrying the SPIONS displayed higher bone mineral density and trabecular volume/tissue volume, supported by greater new bone formation on histology. Interestingly, histology also revealed enhanced blood vessel formation alongside bone development, and it was evident that osteoblasts and vascular endothelial cells had internalised the SPIONs leading to elevated osteogenic and angiogenic performances [19]. In support of these findings, another study reported neo blood vessel formation in addition to bone formation [56] when SPIONS were included in PCL scaffolds. In both these studies, the SPION containing constructs gave rise to substantially greater neovascularisation in comparison to the controls that contained no SPIONs implying potential pro-angiogenic effects. The results from these in-vivo studies are promising, however, the mechanisms by which MNPs promote angiogenesis are yet to be elucidated. Thus, in-vitro studies that investigate the impact of MNPs on endothelial cells are expected to provide insights into the understanding of the angiogenic effects of MNPs and how to best apply them in-vivo for more successful results. However, the pro-angiogenic effect of MNPs is still conflicted as researchers suggest that although it positively impacts osteogenesis, there is no angiogenic activity [60] and furthermore to refute the role of MNPs, a growing body of researchers have discovered the antiangiogenic effect of SPIONs. They are being used to inhibit the growth of tumours by impeding vascular growth. In brief, one in-vitro study reported that polyethyleneimine-coated SPIONs impaired the activation, migration and tube formation of primary human umbilical cord vein endothelial cells (HUVECs). The mechanisms underlying this were attributed to the SPIONs increased reactive oxygen species production that altered actin cytoskeleton activity in HUVECs [67]. Overall, the current research investigating the impact of SPIONs on angiogenesis is conflicting. Some studies have noted a pro-angiogenic impact in BTE, whilst others demonstrate an anti-angiogenic effect in an anti-tumoral therapy investigation, which clearly demonstrates that more exhaustive studies are required.

### 3.3. External Magnetic Stimulation

The application of an external magnetic field can work synergistically to enhance the magnetic stimulation of cells and this combined strategy has been experimented on in BTE, resulting in improved bone formation and an enhanced angiogenic impact than just MNPs alone [68]. The results of a study on a nanocomposite scaffold comprised of PCL/magnetic nanoparticles revealed that the stimulatory effect of the magnetic scaffold and the SMF was more significant than the magnetic scaffold alone [69].

The study showed that the magnetic stimulation equipped the osteoblasts with enhanced functional activity, so they could secrete molecules that have a positive impact on endothelial cell function. This effect was more profound in the combined stimulus group, implying that the addition of an external magnetic field can boost the angiogenic capacity of a magnetic bone scaffold. The effect of this combined strategy was examined to see the effect it had on macrophages [70] and reported that stimulated macrophages could secrete higher levels of angiogenic growth factors compared to unstimulated control cells, which could be attributed to the higher consumption of oxygen by the macrophages because of the more substantial stretching and bending forces within the scaffold from the combined stimulus.

Although studies demonstrate that the application of an external field can enhance osteogenesis and pro-angiogenic potential, the introduction of an external field introduces a handful of complexities. First and foremost, studies should begin to suggest how such a device can be applied clinically with regards to the length of use and follow up. The device should be reasonably practical for the patient, and this should consider parameters such as adherence and ease of manipulation. Importantly, the costs of the magnets should be affordable, given that some strategies in tissue engineering, such as the use of growth factors, have received criticism due to their high costs [64]. Given that all these conditions are met, an external magnetic field can be warranted for use. Alternatively, more research into the use of just MNPs can help determine if the external field is necessary or can be avoided because MNPs alone perform adequately for sufficient angiogenesis.

## 4. Toxicity of Magnetic Nanoparticles

The introduction of MNPs can involve cells internalising them at some stage of the process. Understandably, significant attention has been drawn to the safety of these nanoparticles. An area of research has focussed on the fate of MNPs within the body and more specifically, within cells, to reveal any concerns of toxicity. This section aims to provide a background understanding of the toxicity of MNPs and then assess the role of this in BTE.

### 4.1. Magnetic Nanoparticle-Induced Toxicity

The pharmacokinetics and biodistribution of MNPs is an important consideration that demands exhaustive scrutiny prior to clinical applications. Although SPION injections have been conducted to examine the biodistribution, it has been found to be dependent on the type of SPIONs. SPIONs generally show high levels of accumulation in the kidney as well as organs of the reticuloendothelial system (RES), which includes the liver, spleen and bone marrow [71]. However, this distribution can vary depending on the properties of the MNP under investigation. To exemplify this, a study by Yang et al. [72] reported that carboxyl-coated SPIONs of 10 nm diameter showed the highest uptake by the liver whereas 40 nm nanoparticles were more favoured by the spleen [72], which clearly indicates the size-dependent kinetics of MNPs. Nevertheless, macrophages within the RES internalise SPIONs, where they undergo progressive acid-induced degradation and SPIONs in the kidneys are instead rapidly cleared in the urine [73]. Given this distribution, the impact of SPIONs on these organs have been investigated. A study reflected that at doses of SPIONs above 35 mg/kg, significant toxicity to liver and kidneys occurred, suggesting that toxicity is dose-dependent [74]. In contrast, Pham et al. [75] reported that a dose of 90 mg/kg of SPIONs conjugated to diblock copolymers had no adverse effect on kidney and liver function [75]. Such discrepancies in the literature can be attributed to variation in the features of the SPIONs used by different studies, yielding contrasting findings in the occurrence of toxicity and doses. Nonetheless, it is widely agreed that in-vivo toxicity is dose-dependent, and the non-toxic threshold should be determined for the specific nanoparticle in question. One study noted accumulation in the brain as sufficiently small SPIONs crossed the blood-brain barrier [72], thus it necessitates the modulation of the size of the SPIONs to avoid any impact on brain function. These different studies identify both physical and chemical properties of SPIONs to affect biodistribution and toxicity. These in-vivo studies provide a complex evaluation of the overall effects of SPIONs on a living organism. They can address biological interactions that in-vitro studies cannot, such as the opsonisation of SPIONs and nanoparticle agglomerations within tissues. Finally, the long-term complete clearance of SPIONs is also an area of uncertainty. The clearance of SPIONs is clearly dose-dependent, and higher doses have been demonstrated to take longer to be completely cleared [76,77]. Thus, an in-vivo investigation that monitors subjects for a prolonged period of more than a year is needed to unveil the fate of uncleared MNPs.

A far greater number of in-vitro studies assessing MNP toxicity have been conducted owing to their lower complexity in design, greater control over experimental parameters and easier interpretation [78]. The specific interaction between a particular cell type and MNP can be investigated in detail to gain an insight into cellular and molecular level toxicity. Studies usually involve introducing MNPs to cells in vitro so that the cells internalise the MNPs, resulting in a dose of MNP per cell. It has generally been established that toxicity is again dose-dependent, with high doses eliciting negative effects that reduce cell viability such as cell membrane disruption [79], altered cell cycles [80], reduced motility [81] and genotoxicity [82] to note a few.

Upon internalisation by cells, MNPs are subjected to progressive acid-induced degradation within lysosomes [83]. Degradation of iron oxide-based nanoparticles such as SPIONs releases free ionic iron that is then stored in ferritin protein or alternatively joins the mitochondrial iron pool for cellular use. Besides this, ferroportin can also facilitate the export of ionic iron from the cell, where it is loaded onto transferrin for transport in the bloodstream. Iron-loaded transferrin is endocytosed by target cells that express the transferrin receptor TfR1. To account for increased levels of iron produced from SPION degradation, an adaptive mechanism takes place. Cells upregulate ferritin and ferroportin expression to increase their storage capacity and export excess iron, respectively. Additionally, there is a downregulation of TfR1 expression to limit the uptake of iron from the bloodstream [84].

**Figure 5:**
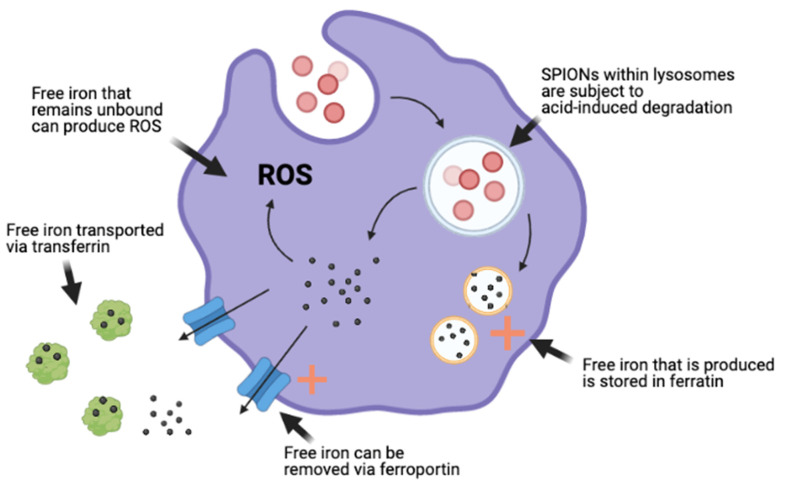
Schematic illustration of the cellular adaptive mechanisms to account for increased free iron produced from SPION degradation, which are upregulated ferratin and ferroportin (indicated by + sign). Diagram also highlights the production of ROS by unbound excess iron.

Cells can thus experience an iron overload from the degradation of a high dose of internalised SPIONs. Their adaptive capacity to handle an increased level of iron is exceeded, resulting in free iron ions within cells. As a part of the Fenton reaction, these unbound ions can react with hydrogen peroxide to produce hydroxyl radicals, a reactive oxygen species (ROS) (Figure 5). These ROS are believed to facilitate the toxic impacts on cells via oxidative stress [85]. Thus, cells are confronted with ROS-mediated toxicity due to high doses of SPIONs [84].

The literature proposes that there are a variety of parameters that can influence the dose of MNP per cell. Both structural features and their coating can influence the cellular interaction of MNPs. Positively charged coatings attach more preferentially to cells, which increases the likelihood of internalisation [86]. Subtle modifications in MNP structure can influence their internalisation potential, hence the cellular MNP dose. Finally, experimental parameters such as incubation, concentration and time can impact the MNP dose internalised by cells [84].

To conclude, cells experience toxicity beyond a certain dose of MNP, however, this toxic dose is cell-specific. Moreover, the features of the chosen MNP can influence the dose cells receive as well as the incubation parameters. Therefore, it is essential that each cell-nanoparticle interaction must be closely examined to avoid toxicity in any therapeutic application.

### 4.2. The Significance of Toxicity on Bone Tissue Engineering Applications

The internalisation of MNPs by stem cells is a recurring theme in BTE applications because of their ability to enhance osteogenic differentiation. Bearing this in mind, MNPs must not cause cytotoxicity nor inhibit osteogenesis this way. As it was concluded that toxicity is dose-dependent and cell-specific, the results of several studies which assessed the impact of dose of SPIONs had on the osteogenic ability of stem cells have been collated and tabulated in Table 2. All the studies followed a similar labelling procedure, involving the incubation of cells in a cell-culture medium containing a given concentration of SPIONs for a given time during which cells internalised the SPIONs [87]. Some studies also estimated the iron content per cell, which is essentially the resultant dose of SPIONs within a cell. To assess the impact of labelling on osteogenic differentiation, cells were then cultured in an osteogenic medium. The data collated in Table 2 aimed to investigate if there was a relationship between the dose of SPION and its impact on the osteogenic potential of stem cells. Upon initial examination, there is no clear trend between the two variables across the studies. For example, one study found that osteogenesis was impaired at 13 pg per cell [88] whereas other studies observed no impact at higher doses of 28 pg [89] and 70 pg [90].

Fan et al. found that the osteogenic potential of BMSCs and ADSCs was decreased when they were labelled with citric-acid-coated SPIONs, with the intracellular iron content being approximately 13 pg [88]. They also conducted a cell viability study, which concluded that viability decreased as the concentration of SPIONs increased. This fall in osteogenic potential and viability was attributed to a high intracellular iron concentration that led to ROS-mediated toxicity as described previously. Although not mentioned in this study, it can be presumed that excess free iron ions were produced from the degradation of the SPIONs, which participated in the Fenton reaction to produce free radicals. However, Andreas et al. who also used citric-acid-coated SPIONs found that an intracellular iron content of 70 pg did not affect the osteogenic potential along with cell viability remaining unimpacted [90]. A key difference between the two studies was that Fan et al. used rat-derived stem cells whereas Andreas et al. used those from humans. It is suspected that human stem cells can store and metabolise a greater quantity of ionic iron before they experience toxicity in comparison to rat stem cells. The fact that rat-derived stem cells exhibited toxicity could indicate that human stem cells may also be similarly affected at a high dose.

Two more studies that studied human stem cells also witnessed an impairment in the osteogenic potential after labelling with SPIONs, although there was no evidence of cellular toxicity alongside [91,92]. One of the studies found that cell viability increased, shown by a higher cell proliferation rate [91] whilst the other noticed that cell migration was promoted in labelled cells [92]. It was suggested that the SPIONs were responsible for altering growth factor release or cell signalling pathways. These alternations inhibited osteogenic differentiation without having a negative impact on cell viability. Therefore, these studies show that osteogenic potential was reduced in the absence of toxicity [91,92]. Furthermore, it was discovered that no toxicity was observed at an intracellular iron content of up to 200 pg, which was the highest dose achieved across the studies included [91]. This indicates that the toxic dose could be beyond this value, however, this is yet to be proven.

It is highly probable that the coating of SPIONs can reduce the risk of toxicity. One study that utilised polydopamine-coated SPIONs found that cell viability remained unaffected at an incubation concentration of 50 μg/mL [93]. As previously mentioned, acid-induced degradation of the SPIONs takes place within lysosomes. The polydopamine coating is a buffered pH-controlled layer. This means that it has a proton sponge effect, by absorbing hydrogen ions within the acidic lysosome environment. As a result, this raises the osmotic stress, which causes the SPIONs to be released into the cytoplasm in a process termed lysosomal escape [94]. Meanwhile, two different studies adopted a silica coating that also preserved cell viability [18,95]. The silica layer provided surface passivation, which meant that the SPIONs were more resistant to lysosomal acidity [94]. These coatings, therefore, help the SPIONs avoid degradation and the subsequent release of iron ions that facilitate ROS mediated toxicity when in excess. Coatings can significantly attenuate the risk of toxicity this way.

Leading on from this, although coatings can be highly protective against degradation, the question of the fate and impact of these SPIONs remains unanswered. Andreas et al. achieved intracellular iron contents per cell of 70 and 26 pg with citrate- and dextran-coated SPIONs, respectively [90]. They reported no cytotoxicity within their study period. If the SPION core along with its coating remains intact, the eventual degradation profile of these SPIONs needs to be studied. It must be determined for how long they resist degradation and the rate at which they degrade if this occurs because this directly affects the rate of iron release within the cell. The fate of undegraded SPIONs is a diverse and complex area of study. Chang et al. found that the iron content per cell decreased with cell proliferation. This suggests that the SPIONs were distributed amongst a greater number of cells, which diluted the intracellular iron content with each cell division [91]. This may alleviate the risk of cellular toxicity. An in-vivo study conducted by Ledda et al. showed that SPIONs accumulated in the liver and the lungs [95], although no tissue damage occurred, the SPIONs were still present within these tissues after 7 weeks. Most studies in Table 2 have shown that SPIONs are not toxic to cells or body tissues and in some cases can promote osteogenic differentiation [18,20,28]. However, their unconfirmed degradation in-vitro and long-term presence in-vivo keeps them under scrutiny.

**Table 2:** A table summarising the various studies that investigated the impact of SPION dose on the osteogenic differentiation of stem cells.

Cell Type	SPION Core-Coating (Name If Given)	SPION Diamete (nm)	SPION Incubation Concentration (μg/mL)	Incubation Period	Iron Content per Cell (pg)	Experiment Duration (Days)	Impact on Osteogenic Differentiation	Other Experiments
Rat BMSCs [88]	Iron oxide- citric acid	96	50	72 h	13	14	Impaired	Reduced cell viability with increasing concentration.
Rat ADSCs [88]	Iron oxide- citric acid	96	50	72 h	13	14	Impaired	Reduced cell viability with increasing concentration.
hMSCs [91]	Magnetite- amine (NH_3_^+^)	6	50	72 h	200	21	Impaired	Improved cell proliferation.
hMSCs [92]	Iron oxide- carboxydextran (Ferucarbotran)	62	100	60 min	NI	7	Impaired	Cell mobilisation was promoted.
hMSCs [95]	Iron oxide- silica	4.5	50	4 days	4	14	Unaffected	Cell viability and proliferation was unimpacted. No changes in gene expression of VEGF or anti-inflammatory factors. No tissue damage or blood toxicity in-vivo after 7 weeks.
hMSCs [88]	Magnetite- citric acid	48	100	72 h	NI	14	Unaffected	Cell viability was unaffected.
Canine ADSCs [89]	Magnetite	10	50	12 h	28	21	Unaffected	Cell viability and proliferation were unimpacted.
hMSCs [93]	Magnetite- PDA	57	50	24 h	NI	21	Unaffected	Cell viability and proliferation were unaffected.
hMSCs [90]	Iron oxide- citrate	98	25	24 h	70	NI	Unaffected	No cytotoxicity was observed.
hMSCs [90]	Iron oxide- dextran (Ferumoxide)	157	500	24 h	26	NI	Unaffected	No cytotoxicity was observed.
hMSCs [20]	Maghemite- PSC	30	100	72 h	NI	21	Promoted	Cell viability was unimpacted.
hMSCs [18]	Magnetite- silica	55	100	24 h	NI	14	Promoted	Cell viability and proliferation were unaffected.
hMSCs [28]	Maghemite- PSC	30	100	48 h	0.9	21	Promoted	Cell viability was unimpacted.

Magnetite—${\mathrm{Fe}}_{3}O_{4}$; Maghemite—${\gamma \mathrm{Fe}}_{2}O_{3}$. Core was referred to as “Iron oxide” if not mentioned in the study. Abbreviations: PDA—Polydopamine, PSC—polyglucose sorbitol carboxymethyl-ether.

## 5. Conclusions and Future Perspectives

To conclude, this review discusses the diverse ways through which MNP can augment BTE, by offering novel solutions and enhancements to each of the three tissue engineering components. Cells experience a marked improvement in osteogenic differentiation and cell proliferation through direct interactions with SPIONs. This is supported by in-vivo findings, showing that osteoblasts and endothelial cells internalise the SPIONs, yielding superior bone regeneration accompanied by blood vessel formation. Moreover, the use of an external magnetic field offers control over MNP labelled cells, providing advances in minimally invasive cell-based regenerative therapy of bone defects and fabricating scaffold-free cell-based constructs for BTE. Furthermore, bioactive agents carrying MNPs can then be delivered to target sites under external magnetic control. Specifically, SPIONs can transport angiogenic plasmids to cells within a scaffold or precisely produce growth factor gradients for complex bone tissue interface engineering. 

Finally, MNP-incorporated scaffolds hold a clear advantage over generic scaffolds attributed to their enhanced mechanical properties and cell performance in-vitro. However, the disagreement in the literature regarding how this occurs needs attention to elucidate the impact. More research investigating the impact of demagnetising MNP-loaded scaffolds on cell performance should be conducted. This will help clarify if the magnetic microenvironment genuinely plays a role in improving cell performance along with the justified effect of the nano structural properties of MNP-incorporated scaffolds. If magnetism does play a role, further research exploring the activation of intracellular pathways is of interest. This research should incorporate a range of stem cell sources to uncover any differences or similarities in the responses between stem cell types. Nevertheless, the enhanced in-vitro cell performances were strongly supported in-vivo, as the scaffolds displayed greater bone regeneration and host tissue integration. However, the literature has only lightly touched upon the enhanced angiogenic performance of the scaffolds in-vivo. Studies investigating the impact of MNP-loaded scaffolds and an external magnetic field on angiogenesis have been conducted, however more studies are required. Future research warrants the study of the impact of solely MNP loaded scaffolds. This could take shape with an in-vitro study, exploring the effect that the scaffold has on endothelial cell performances. What is also evident in the literature is that these enhancements are dose-dependent. Beyond a given dose, agglomeration and potential toxicity of the MNPs may hinder the mechanical properties and cell performance, respectively.

Leading on from this, this review aimed to explore the concerns of toxicity related to MNPs. Both in-vitro and in-vivo studies have evidenced the fact that toxicity is dose-dependent. It has been established that the toxic dose is cell-specific and the MNP features can directly influence the dose that is internalised by cells. Therefore, each specific MNP-cell interaction should be closely examined to prevent toxicity in each biomedical application.

Given these findings, this review collated data from the literature to examine the impact that a given dose of SPION had on the osteogenic differentiation of stem cells. Two studies reported that osteogenesis was impaired in human MSCs, however, toxicity played no role in this. The remainder of the studies observed no concern of toxicity in human stem cells with osteogenesis remaining unaffected or even promoted in some cases. It could be suggested that the toxic dose of SPION in human stem cells undergoing osteogenic differentiation should be determined with a future study. However, considering that osteogenesis was in fact promoted at very low doses (0.9 pg), it may be unnecessary to elucidate this toxic dose. What is a more pressing area of future research is the ultimate fate of SPIONs that avoid degradation. Considering that toxicity is caused by SPION degradation, SPION coatings that are degradation resistant can undoubtedly prevent toxicity. The fate of such SPIONs that remain intact and uncleared from the body for long periods needs to be thoroughly investigated in-vivo. A prospective study should monitor the subjects for the length of time required to finally discover the fate of the SPIONs. Given that this is a safe outcome, MNPs will gain further validation for application in biomedical applications.

## Data Availability

This is a review article and the information is available in literature.
